# Wake-active neurons across aging and neurodegeneration: a potential role for sleep disturbances in promoting disease

**DOI:** 10.1186/s40064-014-0777-6

**Published:** 2015-01-17

**Authors:** Anna L Stern, Nirinjini Naidoo

**Affiliations:** Center for Sleep and Circadian Neurobiology, Perelman School of Medicine, University of Pennsylvania, Philadelphia, USA

**Keywords:** Aging, Neurodegeneration, Sleep, Daytime sleepiness, Fragmentation

## Abstract

Sleep/wake disturbance is a feature of almost all common age-related neurodegenerative diseases. Although the reason for this is unknown, it is likely that this inability to maintain sleep and wake states is in large part due to declines in the number and function of wake-active neurons, populations of cells that fire only during waking and are silent during sleep. Consistent with this, many of the brain regions that are most susceptible to neurodegeneration are those that are necessary for wake maintenance and alertness. In the present review, these wake-active populations are systematically assessed in terms of their observed pathology across aging and several neurodegenerative diseases, with implications for future research relating sleep and wake disturbances to aging and age-related neurodegeneration.

## Introduction

Over the course of healthy human aging, many aspects of sleep are significantly altered. Some of these alterations include decreased slow wave sleep (stages 3 and 4), changes in delta power, decreased homeostatic sleep responses, phase shifts, increased instances of sleep-disordered breathing, periodic limb movements, and sleep and wake fragmentation (Bliwise 1993)(Roenneberg et al. 2004)(Ohayon et al. 2004)(Foley et al. 2007)(Conte et al. 2014). Sleep fragmentation refers to frequent nocturnal awakenings, and wake fragmentation refers to an inability to maintain wakefulness throughout the day – often leading to increased daytime napping. Increased instances of napping among the elderly are often attributed to excessive daytime sleepiness (EDS), which affects approximately 18% of cognitively normal adults aged 65–85 (Jaussent et al. 2012) and is the number one sleep-related predictive factor of a poor quality of life in this population (Reid et al. 2006). Age, independent of overall health, is the third most significant risk factor for EDS (Bixler et al. 2005), but EDS is also an even more prominent feature of almost every common age-related neurodegenerative disease including Alzheimer’s disease (AD) (Merlino et al. 2010)(Bonanni et al. 2005), Parkinson’s disease (PD) (Arnulf and Leu-Semenescu 2009), Amyotrophic Lateral Sclerosis (ALS) (Lo Coco et al. 2011), and Frontotemporal Lobar Degeneration (FTLD) (Bonakis et al. 2014). Depending on the disease in question, varying theories exist to explain the emergence of EDS as a result of the underlying pathology – this is a complex task, given that the causes of EDS emergence in both healthy aging and disease are likely to be multifactorial and include fatigue, boredom, or other psychological factors (Bliwise 1993). However, one intriguing possibility is that a unifying histological feature of neurodegenerative diseases, the disruption and loss of wake-active neurons, is triggered or exacerbated by sleep fragmentation and in turn contributes to the observed daytime sleepiness. This is consistent with a model in which the disrupted sleep that is characteristic of normal aging contributes to the increased likelihood of disease onset in the elderly population.

**Figure 1:**
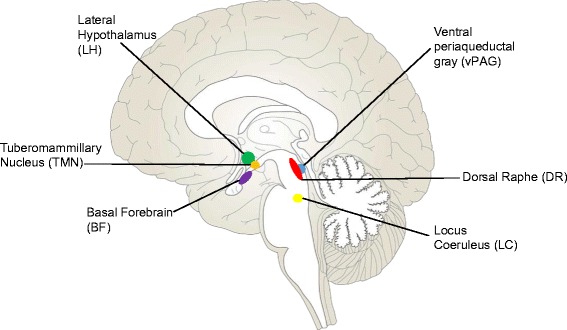
**Midsagittal human brain section showing localization of wake-active brain regions.**

Wake-active neurons are neurons that fire action potentials with high frequency during waking and very low frequency during sleep; these cells include orexinergic (de Lecea and Huerta 2014), noradrenergic (González and Aston-Jones 2006), cholinergic (Platt and Riedel 2011), histaminergic (Huang et al. 2001), serotonergic (Monti 2011), and dopaminergic (Lu et al. 2006) populations (Figure 1). Each of these cell groups is known to be critical for maintenance of consolidated and attentive wakefulness, and each is affected to varying degrees across normal aging and neurodegenerative disease. In this review we aim to synthesize a wide body of literature on the changes observed in these wake-active cells in aging and disease (summarized in Table 1), providing evidence that these changes may both contribute to disease progression and be exacerbated by sleep disturbances.

**Table 1:** **Changes in wake-active neuronal populations across aging and neurodegenerative disease**

**Cell type**	**Region**	**Aging**	**Alzheimer’s (AD)**	**Parkinson’s/Dementia with Lewy Bodies (PD/DLB)**	**Amyotrophic Lateral Sclerosis (ALS)**	**Frontotemporal Lobar Degeneration (FTLD)**
Orexinergic	Lateral Hypothalamus (LH)	•Cell loss(Kessler et al. 2011)(Brownell and Conti 2010)	•Cell loss (Fronczek et al. 2012)	•Cell loss (Thannickal et al. 2007)(Lessig et al. 2010)		•Decreased plasma orexin concentration (Çoban et al. 2013)
		•Loss of fibers (Stanley and Fadel 2012)(Downs et al. 2007)(Zhang et al. 2005)	•Decreased CSF orexin concentration (Fronczek et al. 2012)	•Lewy Bodies (Fronczek et al. 2007)		
				•Decreased CSF orexin concentration in DLB (Wennström et al. 2012)		
		•Decreased responsiveness to orexin in projection areas (Stanley and Fadel 2012)				
Noradrenergic	Locus Coeruleus (LC)	•Decreased noradrenaline reuptake at terminals (Shores et al. 1999)(Zhu et al. 2005)(Shirokawa et al. 2003)	•Cell loss (Brunnström et al. 2011)	•Cell loss (Brunnström et al. 2011)	•Loss of neuron pigmentation (Hoogendijk et al. 1995)	•Cell loss (Brunnström et al. 2011)
		•Decreased DBH (Zhu et al. 2005)	•NFTs (Grudzien et al. 2007)	•Lewy Bodies (Seidel et al. 2014)	•Intracellular inclusion bodies (Iwanaga et al. 1997)	
		•Increased CHOP (Naidoo et al. 2011)				
Cholinergic	Nucleus Basalis of Meynert (NBM)	•Cell loss (Wolf et al. 2014)(Grothe et al. 2012)	•Cell loss (Rogers et al. 1985)	•Cell loss (Rogers et al. 1985)(Grothe et al. 2014)(Iranzo et al. 2014)	•Intracellular inclusion bodies (Matsuoka et al. 2011)	
		•Decreased nicotinic receptor expression in cortex (Nordberg et al. 1992)(Uchida et al. 2013)	•NFTs (Rogers et al. 1985)(Iraizoz et al. 1999)	•Lewy Bodies (Rogers et al. 1985)		
		•Altered AMPA receptor expression(Ikonomovic et al. 2000)				
Histaminergic	Tuberomammillary Nucleus (TMN)	•Elevated levels of CSF histamine metabolites (Prell et al. 1988)	•Cell loss (Nakamura et al. 1993)(Shan et al. 2012a)	•Lewy Bodies (Shan et al. 2012b)		
		•Decreased binding of cortex histamine receptors (Yanai et al. 1992)	•NFTs (Nakamura et al. 1993)	•Increased density of histaminergic fibers in SN (Anichtchik et al. 2000)		
			•Regional alterations in HDC expression (Shan et al. 2012a)			
Serotonergic	Dorsal Raphe (DR)	•Region-specific alterations in 5-HT receptor expression (Rodríguez et al. 2012)(Marcusson et al. 1984)	•Cell loss (Chen et al. 2000)	•Cell loss (Halliday et al. 1990)	•Decreased CSF levels of 5-HT precursor tryptophan(Monaco et al. 1979)	•Cell loss (Yang and Schmitt 2001)
			•NFTs (Chen et al. 2000)	•Lewy Bodies (Seidel et al. 2014)		•Reduced 5-HT1A and 5-HT2A receptor expression in cortex (Bowen et al. 2008)
				•Decreased CSF 5-HT		
			•Decreased CSF 5-HT concentration(Tohgi et al. 1992)	concentration (Tohgi et al. 1993)	Decreased 5-HT receptor expression in cortex (Turner et al. 2005)	
		•Reduced SERT expression (Rodríguez et al. 2012)	•Decreased 5-HT receptor expression in cortex (Lai et al. 2005)	•Reduced 5-HT signaling throughout brain (Politis et al. 2012)		
		•Decreased 5-HT fiber density and aberrant morphology (van Luijtelaar et al. 1988)				
Dopaminergic	Ventral Periaqueductal Gray (vPAG)		•NFTs (Parvizi et al. 2000)	•Cell loss (Benarroch et al. 2009)		
				•Lewy Bodies (Seidel et al. 2014)(Benarroch et al. 2009)		
				•Increased rates of dopamine metabolism (Kumakura et al. 2010)		

## Sleep phenotypes across neurodegenerative disease

### Alzheimer’s disease

Alzheimer’s Disease (AD) is the most common age-related neurodegenerative disease in the world, and it is the leading cause of dementia (Peter-Derex et al. 2014). Brains of AD patients accumulate aggregates of β-amyloid and hyperphosphorylated tau called plaques and neurofibrillary tangles (NFTs), respectively (Bloom 2014). This pathology is accompanied by widespread neuronal loss, particularly in cortical and subcortical regions involved with cognition and memory (Bouras et al. 1994). Up to 45% of Alzheimer’s patients suffer from at least one sleep disorder (Peter-Derex et al. 2014). Nocturnal awakenings and EDS are the most common of these, with one study finding that the average AD patient gets over 14% of their total sleep during the daytime (Vitiello et al. 1992). The extent to which sleep is fragmented is correlated with severity of dementia (Pat-Horenczyk et al. 1998), and it is one of the leading causes of institutionalization among patients (Bonanni et al. 2005). Although sleep consolidation does decline as the disease progresses and is often considered to be a consequence of disease, there is retrospective evidence that frequent daytime napping in healthy elderly subjects is predictive of a later diagnosis of AD in those carrying the ApoE-ε4 allele, which confers genetic risk of AD (Lim et al. 2013b). This could indicate that either EDS itself or loss of integrity of wake-active neurons is a contributing factor in the onset of AD.

### Parkinson’s disease

Parkinson’s Disease (PD), the second most common neurodegenerative disease, is characterized primarily by a loss of movement and postural control resulting from decreased dopaminergic neurons in the area substantia nigra (SN). Degeneration in SN and other areas is accompanied by accumulation of Lewy Bodies, intracellular aggregates of α-synuclein (Langston et al. 2013). Almost all PD patients experience diminished sleep quality of some kind (Lima 2013), and the namesake of the disease James Parkinson even noted in his first published description of symptoms that “…the sleep becomes much disturbed” (Parkinson 2002). PD patients are awake for an average of 30-40% of the night (De Cock et al. 2008), and over a third of patients experience EDS during the day (Arnulf and Leu-Semenescu 2009) – this is reflected by a 225% increase in time spent napping by PD patients as compared with age-matched controls (Bolitho et al. 2013). In one study, elderly subjects with EDS had more than three-fold higher likelihood of receiving a future diagnosis of PD (Abbott et al. 2005), indicating that disturbed sleep/wake may be a contributing factor rather than a consequence of disease.

PD also has high comorbidity with Rapid Eye Movement Sleep Behavior Disorder (RBD) (Gong et al. 2014), a parasomnia characterized by the loss of normal paralysis during rapid eye movement (REM) sleep, causing patients to physically act out their dreams. Although the pathophysiology of this disorder is not well understood, animal studies and post mortem analysis reveal a likely role for the breakdown of pontine brain areas involved in regulating sleep cycles (Boeve et al. 2007). One study found that over 56% of PD patients met the criteria for RBD diagnosis (Gong et al. 2014), and idiopathic RBD is also a very reliable predictor of later PD development. A recent update on a ten year longitudinal study reported that over 80% of RBD patients assessed, who showed no other signs of neurodegenerative disease at the time, received an eventual diagnosis of PD or other synucleinopathies such as Dementia with Lewy Bodies (DLB) (Schenck et al. 2013). The strong predictive value of RBD, like EDS, indicates a possible role for sleep disruption in PD onset.

### Amyotrophic lateral sclerosis

In patients with Amyotrophic Lateral Sclerosis (ALS), degeneration of lower motor neurons leads to muscle weakness, paralysis, and eventual death (Rowland 2010). Up to 50% of these patients report difficulty staying asleep at night, indicating that their sleep is fragmented (Lo Coco et al. 2011). In ALS in particular, EDS is often referred to more broadly as fatigue, which affects 40-80% of patients and is strongly correlated with both disease severity and depression (Lo Coco and La Bella 2012). It is unclear in ALS to what extent fatigue is a result of poor sleep quality or a direct consequence of motor neuron degeneration, but evidence suggests that sleep complaints are highly correlated with degree of sleepiness (Lo Coco and La Bella 2012), suggesting that sleep plays a role.

### Frontotemporal lobar degeneration

Frontotemporal Lobar Degeneration (FTLD), a broad term encompassing a wide range of pathologies that all involve degeneration of the frontotemporal region, accounts for 10% of all instances of dementia (Karageorgiou and Miller 2014). Circadian rhythms are severely disrupted in FTLD patients (Anderson et al. 2009), and one study found that 64% of these patients suffer from EDS (Guarnieri et al. 2012). Recently, physicians observed that FTLD is characterized by even more severe sleep symptoms than AD, and that the onset of symptoms occurs very rapidly during the course of disease (Bonakis et al. 2014).

## Wake-active neuronal pathology across aging and neurodegenerative disease

Given the surprising consistency of sleep and wake disturbances, particularly EDS, across normal aging and various neurodegenerative diseases, a careful consideration of the potential role played by wake-active neurons in disease onset and progression is warranted. Consistent with sleep symptoms, all wake-active populations indeed undergo drastic changes over the course of aging, which may be both a cause and consequence of declining sleep quality.

### Orexinergic neurons of lateral hypothalamus

A small population of cells in the lateral hypothalamus (LH) releases the neuropeptides orexin-A/hypocretin-1 and orexin-B/hypocretin-2, which coordinate wakefulness and alertness through direct communication with other wake active brain areas including locus coeruleus (LC), tuberomammillary nucleus (TMN), dorsal raphe nucleus (DRN), and ventral periaqueductal gray (vPAG) (de Lecea et al. 1998)(Peyron et al. 1998). Loss of orexinergic neurons in both humans (Thannickal et al. 2000) and animals (Chemelli et al. 1999)(Lin et al. 1999) results in a narcoleptic phenotype characterized by an inability to maintain wakefulness. Optogenetic stimulation of these neurons increases the probability of a transition from sleep to wake (Adamantidis et al. 2007), and their silencing induces sleep (Tsunematsu et al. 2011).

Consistent with a loss of wake consolidation with aging (Foley et al. 2007), substantially decreased numbers of orexin neurons have been observed in aged rats (Kessler et al. 2011) and mice (Brownell and Conti 2010), and the remaining neurons have decreased signs of activation following sleep deprivation (Naidoo et al. 2011). Moreover, orexin signaling in downstream wake-active regions is diminished in several animal models. One group of researchers found that in aged rats, orexinergic fibers projecting to hippocampus are decreased in correlation with blunted cholinergic release in response to orexin (Stanley and Fadel 2012). Innervation of LC by orexinergic projections is decreased as well in aged rhesus macaques (Downs et al. 2007), and similar data were obtained in the basal forebrain of guinea pigs (Zhang et al. 2005). Considered together, these studies indicate that the orexin system undergoes widespread changes in both size and functionality over the course of aging, which is likely to contribute to EDS in the elderly.

In addition to changes in orexinergic function affecting sleep, decreases in sleep may in turn affect the orexin system. Chronic sleep fragmentation, for instance, decreases activation of orexinergic neurons in response to hypercapnia and decrease orexinergic projections to the cingulate cortex (Li et al. 2013). Furthermore, acute sleep deprivation causes both an increased sensitivity of orexinergic neurons to the inhibitory neurotransmitter GABA (Matsuki et al. 2014) and a switch from excitation to inhibition in response to noradrenergic signaling (Grivel et al. 2005). One recent study found that when mice were deprived of sleep for 12 hours a day over 7 days, 24% of orexinergic cells in lateral hypothalamus were lost (Obukuro et al. 2013). This neuronal loss was dependent on S-linked nitrosylation of the critical foldase protein disulfide isomerase (PDI), and much evidence suggests that this particular protein modification plays a critical role in neurodegenerative diseases associated with protein misfolding (Uehara et al. 2006)(Halloran et al. 2013). Thus, one possibility is that sleep loss exacerbates the age-related changes in orexinergic neurons through protein dyshomeostasis, eventually leading to the development of a cellular environment that is highly susceptible to neurodegeneration (Brown and Naidoo 2012)(Roussel et al. 2013). Consistent with this, AD, PD, and DLB patients have decreased orexin cell numbers compared with age-matched controls (Fronczek et al. 2012)(Thannickal et al. 2007)(Lessig et al. 2010), and FTLD patients have decreased levels of orexin-A (Çoban et al. 2013).

### Noradrenergic neurons of locus coeruleus

The LC serves a wide range of functions relating to autonomic activity, stress, learning, and arousal. The area sends dense noradrenergic projections throughout the cortex and other brain regions, including excitatory inputs to wake-active nuclei and inhibitory inputs to sleep-promoting centers such as the ventrolateral preoptic area (VLPO) (Samuels and Szabadi 2008). Locus coeruleus neurons are active during wake, particularly alert wakefulness, and are silent during sleep (Takahashi et al. 2010). Recently, the role of LC in modulating sleep-wake state was shown using optogenetic manipulations; in these experiments, stimulating noradrenergic LC neurons caused a transition from sleep to wakefulness, while silencing the same population induced sleep (Carter et al. 2010).

It has become clear over the past two decades that LC neuronal number is preserved in healthy aging (Mouton et al. 1994)(Ohm et al. 1997). However, data from both humans and animals indicate that the connectivity, expression patterns, and function of these neurons are altered over time. In humans, LC neuromelanin content increases in middle age and decreases in the elderly, which may affect susceptibility of neurons to oxidative insults (Shibata et al. 2006). Several studies in rats have demonstrated that over the course of aging, norepinephrine reuptake at axon terminals is decreased in cortex, along with decreased levels of norepinephrine transporter (NET) mRNA (Shores et al. 1999)(Zhu et al. 2005)(Shirokawa et al. 2003). Levels of mRNA encoding dopamine β-hydroxylase (DBH), the enzyme catalyzing the conversion of dopamine to norepinephrine, also decline in LC with age (Zhu et al. 2005). In LC of aged mice, expression of the pro-apoptotic factor C/EBP homologous protein (CHOP), which promotes cell death in response to protein dyshomeostasis, is dramatically increased (Naidoo et al. 2011).

Similarly to the case of orexinergic neurons, evidence in animals suggests that LC is highly susceptible to sleep disturbances. In cats, REM sleep deprivation during postnatal day 42–49 causes over half of LC neurons to be lost, in addition to an overall decrease in size of remaining cells (Shaffery et al. 2012). Mouse LC neuron number is decreased following both intermittent hypoxia – interruptions in breathing experienced in sleep apnea – and chronic sleep deprivation (Zhu et al. 2007)(Zhang et al. 2014). Following sleep deprivation for 8 hours/day for 3 days, the critical redox homeostasis protein SirT3 is downregulated in LC neurons, and this is associated with increased oxidative stress and a 20% neuronal loss (Zhang et al. 2014). Reasons for LC susceptibility to stressors such as sleep loss are unclear, but various hypotheses have been proposed. High levels of NADPH oxidase may play a role in contributing to oxidative injury (Zhu et al. 2007)(Zhan et al. 2005), and recent evidence from slice recordings demonstrates that LC neurons experience augmented mitochondrial oxidant stress due to basal calcium oscillations (Sanchez-Padilla et al. 2014).

In addition to its vulnerability to alterations in the sleep-wake cycle, LC appears to be uniquely vulnerable in neurodegenerative disease (Sotiriou et al. 2010)(Von Coelln et al. 2004). The majority of LC noradrenergic neurons are lost in AD, PD, and DLB (Brunnström et al. 2011), to a lesser extent in FTLD (Brunnström et al. 2011), and morphological and histological changes occur in LC of ALS patients as well (Hoogendijk et al. 1995)(Iwanaga et al. 1997). Recently, it was reported that neuron loss in LC of AD and PD patients is even greater than that observed in regions of the forebrain and substantia nigra, respectively (Zarow et al. 2003). Anatomist Heiko Braak has written extensively on his findings regarding the stages of the pathological process in both AD and PD, and in both cases it is clear that LC pathology occurs long before most other regions incur damage (Braak et al. 2011)(Braak et al. 2004)(but see (Burke et al. 2008) for an alternative view). Suggesting a causal role for this early LC degeneration, toxic lesions of LC in a transgenic mouse model of Alzheimer’s disease accelerate β-amyloid plaque formation, acetylcholinesterase activity reduction, neuronal loss, and onset of memory impairment (Heneka et al. 2006).

The view that early LC degeneration may cause further disease progression is also bolstered by an extensive study compiling cognitive and histological data from 165 elderly individuals without a prior diagnosis (Wilson et al. 2013). Researchers collected data using a battery of cognitive tasks spanning several years, and upon death the subjects’ brains were autopsied. In addition to neuronal size and number, researchers quantified neurofibrillary tangles and Lewy Bodies. They found that the presence of these pathological hallmarks in LC, but not SN or other brain regions examined, was strongly correlated with cognitive decline. Based on these data, the authors concluded that neuronal health in LC may determine whether damage to other brain regions will result in neurological symptoms (Wilson et al. 2013). In this scenario, declining function of LC neurons due to sleep fragmentation could accelerate disease progression or even allow new symptoms to emerge as a result of existing degeneration in other brain regions.

Based on the strong correlations between sleep fragmentation, neurodegenerative disease, and LC neuronal loss, it is logical to infer that as sleep patterns change with age, vulnerable noradrenergic neurons of the LC may lose functionality (Zhang et al. 2014) and in turn promote disease onset or progression (Wilson et al. 2013)(Braak et al. 2004). This framework is consistent with EDS being predictive of declines in cognition (Jaussent et al. 2012) and the sleep disorder RBD being the most robust known predictor of PD (Schenck et al. 2013). This also would explain the pathological timeline of disease (Braak et al. 2011)(Braak et al. 2004) and provide a potential contributing explanation for why neurodegeneration occurs most commonly in the elderly, whose sleep is highly fragmented (Schmidt et al. 2012).

### Cholinergic neurons of nucleus basalis of Meynert

A cluster of neurons in the basal forebrain (BF) called the Nucleus Basalis of Meynert (NBM) provides the primary source of cholinergic input to regions throughout the cortex (Gratwicke et al. 2013). NBM is critically involved in cognition (Hasselmo and Sarter 2011), wakefulness (Manfridi et al. 1999), and REM sleep (Steriade 2004), receiving dense projections from other wake-active areas such as LC and TMN (Platt and Riedel 2011).

Atrophy of NBM and other cholinergic nuclei occurs in healthy aging (Grothe et al. 2012)(Wolf et al. 2014)(Sawiak et al. 2014) (but see (Schliebs and Arendt 2011) for an alternative view), along with a drastic reduction in nicotinic acetylcholine receptor expression in cortex (Nordberg et al. 1992). Moreover, existing cholinergic cells may not be as responsive to environmental stimuli (Zhang et al. 2002); this could be due to a number of changes in protein expression patterns, including loss of AMPA receptors (Ikonomovic et al. 2000). Based on the critical role of cholinergic signaling in learning and memory, it is not surprising that basal forebrain volume is correlated with cognitive ability across aging (Wolf et al. 2014).

In rodent models, both sleep deprivation and fragmentation have marked effects on BF. (Kim et al. 2013)(Sims et al. 2013). After 6 hours of sleep deprivation, *in vitro* studies reveal an inducible nitric oxide synthase (iNOS)-dependent increase in adenosine release from BF neurons (Sims et al. 2013), and *in vivo* studies indicate that increases in iNOS expression occur specifically in wake-active neurons of NBM (Kalinchuk et al. 2010). This indicates that protein nitrosylation may play a role similarly to the case of orexinergic neurons (Obukuro et al. 2013). Additionally after 6 hours of sleep deprivation adenosine receptor expression is upregulated in BF (Basheer et al. 2007), and after 24 hours of sleep deprivation, levels of α1-adrenergic receptor mRNA in BF are increased as well (Kim et al. 2013).

Similarly to other wake-active regions, NBM neuron numbers are also substantially decreased in neurodegenerative disease, primarily AD (Rogers et al. 1985), PD, (Rogers et al. 1985) and DLB (Grothe et al. 2014)(Iranzo et al. 2014) (although cholinergic systems seem to be uniquely spared in FTLD (Hirano et al. 2010)(Di Lazzaro et al. 2006)). Particularly in AD, selective pathology in cholinergic cells in NBM is an early and defining feature of disease that progresses slowly throughout aging, mild cognitive impairment (MCI), and eventually the first stages of AD (Mesulam et al. 2004). Degeneration of these neurons is likely to play a key role in the progression of symptoms, given that cognition in AD is correlated with BF volume (Grothe et al. 2014), and lower NBM volumes are predictive of cognitive decline in patients with mild cognitive impairment (Grothe et al. 2010). Based on these data as well as the benefit provided by acetylcholinesterase inhibitors for AD patients (Zemek et al. 2014), deep brain stimulation of NBM has recently gained popularity among scientists as a potential therapeutic intervention (Gratwicke et al. 2013). If these strategies prove effective, one implication would be that preservation of NBM integrity through interventions aimed at sleep consolidation could also help to ameliorate disease.

### Histaminergic neurons of tuberomammillary nucleus

The TMN in the hypothalamus is the sole source of the wake-promoting neurotransmitter histamine. The region projects widely throughout the brain and plays a critical role in maintaining circadian rhythms, with direct reciprocal connections to the master circadian clock region suprachiasmatic nucleus (SCN) (Shan et al. 2013). The importance of histaminergic signaling was recently highlighted with the discovery that the effects of orexin on wakefulness are entirely dependent on downstream histamine release (Huang et al. 2001).

Several changes occur in TMN and histamine signaling with aging, although the number of cells is essentially preserved (Shan et al. 2013). Elevated levels of histamine metabolites were identified in the CSF of older subjects (Prell et al. 1988), and decreased expression of histamine receptors in cortex was identified by PET scan (Yanai et al. 1992). This could indicate that an overactive histaminergic system induces receptor downregulation, which would be consistent with reports of increased cell size in TMN of older men (Ishunina et al. 2003). However, neither metabolic activity nor expression of histamine synthesizing enzyme histidine decarboxylase (HDC) are altered over the course of aging (Ishunina et al. 2003) (Shan et al. 2013). Based on these somewhat contradictory data, it is likely that age-related changes to the histaminergic system are relatively subtle and complex.

Histamine concentrations in CSF are decreased in patients with EDS (Bassetti et al. 2010), indicating that low histamine may either contribute to sleepiness or be induced by sleep-wake fragmentation. Supporting the latter possibility, sleep deprivation in rats causes a decline in brain histamine levels (Xu et al. 2010). A loss of orexin, however, which results in instability of sleep-wake states, is associated with dramatic increases in histaminergic neuron number in both humans and mice (Valko et al. 2013).

The histaminergic system is substantially affected in both AD and PD. In AD in particular, dramatic cell loss occurs in TMN (Nakamura et al. 1993)(Shan et al. 2012a) as well as decreased histamine synthesis (Fernandez-Novoa 2001). However, this is accompanied by increased histamine release at axon terminals (Fernandez-Novoa 2001), which may partially compensate for loss of soma. Interestingly, in PD there is increased arborization of histaminergic terminals as well, particularly in the SN (Anichtchik et al. 2000), but despite the extensive spread of Lewy bodies throughout TMN there is no observed loss of cells or HDC expression (Shan et al. 2012b)(Shan et al. 2013). Presynaptic histamine receptor antagonists, which further increase histamine release, are currently in clinical trials to assess their potential efficacy in mitigating symptoms of both AD (Brioni et al. 2011) and PD (Shan et al. 2013).

### Serotonergic neurons of dorsal raphe

The dorsal raphe (DR) nuclei synthesize the neurotransmitter serotonin (5-HT) and send extensive projections through the telencephalon, brainstem, and cortex. The DR receives inputs from all other wake active neuronal populations (Rodríguez et al. 2012) and plays a critical role in maintaining wakefulness (Monti 2011).

A decrease in 5-HT receptor expression has been reported in healthy aging, but contradictory data have been obtained depending on the organism, brain region, and receptor subtype being studied (Rodríguez et al. 2012)(Marcusson et al. 1984). In addition, aged rats have altered serotonin transporter (SERT) expression, aberrant DR neuronal morphology, and decreased fiber density of 5-HT neurons (Rodríguez et al. 2012)(van Luijtelaar et al. 1988). Despite these changes, however, absolute cell number in DR is well preserved over aging in both rats (van Luijtelaar et al. 1992) and humans (Klöppel et al. 2001).

The effects of acute sleep deprivation and REM sleep deprivation on DR have been well studied, in part because sleep deprivation has high therapeutic efficacy in depressed patients, who are thought to have altered 5-HT signaling (Hemmeter et al. 2010). In line with this, animal studies have indeed revealed extensive changes to DR following sleep deprivation, including increases in neuronal size (Ranjan et al. 2010), increased firing during wake (Gardner et al. 1997), and what may be a compensatory downregulation of 5-HT receptors throughout downstream brain regions (Hipólide et al. 2005).

Despite relative preservation of cell number in healthy aging, extensive DR cell loss occurs in AD (Chen et al. 2000), PD (Halliday et al. 1990), and FTLD (Yang and Schmitt 2001) (although the latter data are controversial – see (Rodríguez et al. 2012)), and in all three diseases (Lai et al. 2005)(Politis et al. 2012)(Bowen et al. 2008), as well as in ALS (Turner et al. 2005), reduced 5-HT receptor expression in cortex has also been documented. Reduced CSF concentrations of either 5-HT or its precursor tryptophan are observed in AD (Tohgi et al. 1992), PD (Tohgi et al. 1993), and ALS (Monaco et al. 1979) patients as well. In PD, these decreases in 5-HT concentration are correlated with the severity of motor symptoms (Tohgi et al. 1993), and in AD loss of 5-HT receptor expression in temporal cortex is correlated with cognitive decline (Lai et al. 2005). These data indicate that loss of serotonergic signaling may play a role in disease progression, which is consistent with the observations that DR pathology occurs very early in the development of PD (Braak et al. 2004) and that treatment with selective serotonin reuptake inhibitors (SSRIs) has shown promise in ameliorating AD cognitive symptoms (Mossello et al. 2008). ALS disease progression is likely to be influenced by altered serotonin systems as well, given that motor neurons with dense serotonergic input are preferentially susceptible to degeneration (Sandyk 2006).

### Dopaminergic neurons of ventral periaqueductal gray

The most recently identified wake-active neuronal population is a small group of dopaminergic cells just lateral to the DR called the ventral periaqueductal gray (vPAG). These cells have strongly increased c-fos expression during wakefulness, and their depletion causes an increase in total sleep time in rats (Lu et al. 2006).

Very little has been reported regarding changes to the vPAG during healthy aging, despite extensive attention paid to other dopaminergic systems such as SN and VTA. Based on its recently discovered role in sleep-wake cycle maintenance (Lu et al. 2006), future studies may address whether alterations to this region over aging occurs in association with sleep disturbances. There is also a paucity of data regarding effects of sleep disturbances on vPAG, although one study suggests that vPAG may be uniquely susceptible to intermittent hypoxia (Zhu et al. 2007). In this study, long term intermittent hypoxia induced high levels of the apoptotic marker cleaved caspase 3, as well as cell loss, in dopaminergic cells of vPAG but not orexinergic or histaminergic populations (Zhu et al. 2007). However it is unclear whether these changes were due to sleep fragmentation or hypoxia *per se*.

Regarding disease, loss of vPAG cells (Benarroch et al. 2009) and altered dopamine metabolism (Kumakura et al. 2010) have been reported in PD patients, and vPAG of AD patients (Parvizi et al. 2000) and mouse models of AD (Overk et al. 2009) display extensive NFT and β-amyloid pathology. Taken together, these studies suggest the possibility of a role for vPAG disturbances in disease, but a larger body of evidence is warranted to clarify whether vPAG pathology represents a unique phase of degeneration or a more generalized feature of widespread neuronal damage.

## Conclusion

Although it is widely documented that sleep fragmentation and EDS are strongly correlated with both aging and neurodegenerative disease, it has proven difficult to define the causal relationships among these features, and the topic remains controversial (Klerman and Dijk 2008)(Gooneratne and Vitiello 2014). One recent human study (Mander et al. 2013) elucidated a role for slow wave sleep loss in the memory retention deficits associated with healthy aging, and found that both factors were associated with atrophy of medial prefrontal cortex. The authors concluded that age-associated cortical atrophy may contribute to sleep changes which in turn impact memory, indicating that interventions aimed at improving sleep among the elderly may have marked benefits on cognitive function even in healthy patients (Miyata et al. 2013).

Several animal studies have now provided plausible mechanistic bases for effects of sleep disturbance on neurodegenerative disease onset or progression as well. For instance, it was recently reported that in mice, interstitial fluid levels of β-amyloid are increased with both orexin administration and sleep deprivation (Kang et al. 2009), and one of the key functions of sleep may be to allow clearance from the brain of potentially toxic species including β-amyloid (Xie et al. 2013). Given such studies as well as the breadth of data indicating that loss of sleep and wake consolidation often precedes and predicts neurodegenerative disease (Schenck et al. 2013)(Abbott et al. 2005)(Lim et al. 2013a), scientists should now address whether non-pharmacological (Wennberg et al. 2013) or pharmacological (Wennberg et al. 2013)(Lyseng-Williamson 2012) sleep therapies can decrease the likelihood of disease onset through preservation of wake-active neuronal systems in the elderly population. Indeed, the sleep-promoting hormone melatonin has been shown not only to increase sleep and improve daytime alertness (Lemoine et al. 2007), but also to improve cognitive scores in AD patients (Wade et al. 2014). Cell culture, animal, and human data indicate that melatonin may stall disease progression in ALS patients as well (Weishaupt et al. 2006).

Each neurodegenerative disease exhibits unique pathology, symptomology, genetic risk factors, and environmental correlates, but sleep disturbances are the one feature common across a wide range of diseases. This highlights a role for sleep in neurodegenerative onset as one of the most parsimonious explanations for emergence of disease, and the failure of wake-active neuronal populations following prolonged sleep disruption provides a mechanistic framework to bolster the likelihood of such a model. As the sleep and neurodegeneration fields begin to foster greater collaboration, we expect to see more studies in both animals and humans to determine whether improving sleep may ameliorate the high disease risks faced by the elderly population.
